# DMRTA2 supports glioma stem-cell mediated neovascularization in glioblastoma

**DOI:** 10.1038/s41419-024-06603-y

**Published:** 2024-03-20

**Authors:** Marta Maleszewska, Kamil Wojnicki, Jakub Mieczkowski, Sylwia K. Król, Karol Jacek, Magdalena Śmiech, Marta Kocyk, Iwona A. Ciechomska, Mateusz Bujko, Janusz Siedlecki, Katarzyna Kotulska, Wiesława Grajkowska, Małgorzata Zawadzka, Bozena Kaminska

**Affiliations:** 1 Laboratory of Molecular Neurobiology, Nencki Institute of Experimental Biology, Polish Academy of Sciences, Warsaw, Poland; 2 Department of Animal Physiology, Institute of Functional Biology and Ecology, Faculty of Biology, University of Warsaw, Warsaw, Poland; 3 3P-Medicine Laboratory, Medical University of Gdansk, Gdansk, Poland; 4 Department of Molecular and Translational Oncology, Maria Sklodowska-Curie National Research Institute of Oncology, Warsaw, Poland; 5 Department of Pathology, The Children’s Memorial Health Institute, Warsaw, Poland; 6 Laboratory of Neuromuscular Plasticity, Nencki Institute of Experimental Biology, Warsaw, Poland

**Keywords:** Cancer stem cells, Tumour angiogenesis

## Abstract

Glioblastoma (GBM) is the most common and lethal brain tumor in adults. Due to its fast proliferation, diffusive growth and therapy resistance survival times are less than two years for patients with IDH-wildtype GBM. GBM is noted for the considerable cellular heterogeneity, high stemness indices and abundance of the glioma stem-like cells known to support tumor progression, therapeutic resistance and recurrence. Doublesex- and mab-3–related transcription factor a2 (DMRTA2) is involved in maintaining neural progenitor cells (NPC) in the cell cycle and its overexpression suppresses NPC differentiation. Despite the reports showing that primary GBM originates from transformed neural stem/progenitors cells, the role of DMRTA2 in gliomagenesis has not been elucidated so far. Here we show the upregulation of *DMRTA2* expression in malignant gliomas. Immunohistochemical staining showed the protein concentrated in small cells with high proliferative potential and cells localized around blood vessels, where it colocalizes with pericyte-specific markers. Knock-down of DMRTA2 in human glioma cells impairs proliferation but not viability of the cells, and affects the formation of the tumor spheres, as evidenced by strong decrease in the number and size of spheres in in vitro cultures. Moreover, the knockdown of DMRTA2 in glioma spheres affects the stabilization of the glioma stem-like cell-dependent tube formation in an in vitro angiogenesis assay. We conclude that DMRTA2 is a new player in gliomagenesis and tumor neovascularization and due to its high expression in malignant gliomas could be a biomarker and potential target for new therapeutic strategies in glioblastoma.

## Introduction

Malignant gliomas (WHO grades 3 and 4) are the most common primary tumors of the central nervous system (CNS) in adults [1]. Glioblastoma (GBM, classified as a WHO grade 4) due to its aggressiveness, highly diffusive growth and vascularization is a deadly tumor with average survival of patients less than 15 months [2]. The intratumoral cellular heterogeneity and diffusive growth of GBM make complete surgical resection difficult. Particularly, the presence of tumor stem-like cells contributes to therapeutic resistance and quick tumor recurrence after surgery combined with radio and chemotherapy [3].

Due to their fatality and limited therapeutic options, malignant gliomas are intensively studied to identify biomarkers and pathological mechanisms. The numerous studies have characterized different glioma groups and subtypes with regard to DNA mutations, DNA copy number alterations, gene expression, DNA methylation, miRNA or protein expression profiles [4, 5]. Common oncogenic drivers of pediatric and high and lower grade adult gliomas were found [6], but up to now standard and personalized therapies were not effective. Intensive attempts at understanding the underlying pathobiology are undertaken to find new targets that would expand therapeutic options for GBM patients.

Doublesex and mab-3-related transcription factor a2 (Dmrta2) is the DM domain transcription factor involved in gonadal differentiation and CNS development. The expression of Dmrta2/DMRTA2 in neural tissues during development, and its role in neurogenesis and normal brain development have been documented in Zebrafish [7], Xenopus [8], mice [9–12] and humans [13]. Studies of Dmrta2 null mice indicate that the genetic ablation of *Dmrta2* causes the hypoplasia or loss of the medial structures in the telencephalic cortex, thus implying that Dmrta2 plays a pivotal role in the regulation of neocortical patterning and in the formation of the signaling center for brain differentiation [7, 9]. In zebrafish, Dmrta2 controls *neurogenin1* expression by repressing *her6* in the posterior-dorsal telencephalon and the lack of Dmrta2 function affects the neuronal differentiation gene—*neurod* expression [7]. In mouse, Dmrta2 plays pivotal roles in the early development of the telencephalon and in the maintenance of neural progenitors as a downstream component of the Wnt pathway [9]. Loss of Dmrta2 results in decreased Wnt and Bmp expression in the dorsomedial telencephalon and in a reduction of the number of Cajal–Retzius cells [14]. Furthermore, Dmrta2 is known as a regulator of the neuron-glia cell-fate switch in the developing hippocampus. Dmrta2 reciprocally regulated Lhx2: loss of either factor promoted gliogenesis; overexpression of either factor suppressed gliogenesis and promoted neurogenesis; while each could substitute for the loss of the other [15]. Moreover, Dmrta2 plays an important role in maintaining neural precursor cells (NPC) in the cell cycle. Loss of Dmrta2 accelerates the cell cycle exit and differentiation of NPCs into postmitotic neurons, while its forced expression suppresses NPC differentiation [16]. Compelling evidence from studies in animal models indicates that neural stem or precursor cells accumulating genetic alterations are glioma-initiating cells and may contribute to the biological and genomic phenotypes of glioblastoma [17]. Nevertheless, to date, there is no data evaluating the role of DMRTA2 in gliomagenesis.

Here, we report that *DMRTA2* is highly upregulated in human gliomas, in particular in WHO grades 3 and 4 gliomas and its expression inversely correlated with patient survival. The protein is detected around blood vessels and in a subset of small tumor cells with high proliferative potential. DMRTA2 expression is also elevated in glioma cell lines compared to normal human astrocytes and is even higher in glioblastoma-derived spheres enriched for cancer stem cells. Knock-down of DMRTA2 in human glioma cells impairs their proliferation without affecting cell viability and reduces formation of tumor spheres in in vitro cultures. Moreover, DMRTA2-depleted glioma spheres demonstrate reduced pro-angiogenic competences and faster tube disruption in an in vitro tube formation assay. Altogether, the results show an important role of DMRTA2 in the maintenance of the stemness phenotype of glioma cells and aberrant angiogenesis.

## Materials and methods

### RNA and DNA purification from glioma samples

The glioma biopsies were obtained from the Canadian Brain Tumor Tissue Bank in London (London Health Sciences Centre, Ontario CA), Children’s Memorial Health Institute, Warsaw, Poland and The Maria Sklodowska-Curie Memorial Cancer Center and Institute, Warsaw, Poland and informed consents were obtained from all subjects. The use of patient tissues was permitted by the Ethical Committee of the respective hospitals.

Total RNA was isolated using Tri Reagent extraction from snap-frozen tissues followed by the RNeasy Mini Kit isolation as previously described [18]. RNA quantity, quality and integrity were verified using the NanoDrop 2000 spectrophotometer (Thermo Scientific) and the Agilent Bioanalyzer 2100 and RNANanoChip assay (Agilent Technologies). DNA precipitation from the interphase and organic phase was performed with 100% ethanol. Samples were centrifuged at 5000*g* for 10 min at 4 °C. After 3 washes in 0.1 M trisodium citrate, 10% ethanol for 30 min and a final wash in 75% ethanol the DNA pellet was air dried and resuspended in H_2_O.

### TCGA data analysis and Kaplan-Meier survival analyses

*DMRTA2* expression within TCGA data was performed in GlioVis—data portal for visualization and analysis of brain tumor expression datasets (http://gliovis.bioinfo.cnio.es). Statistical analysis was performed with Tukey’s Honest Significant Difference (HSD) test. Survival plots were generated in GlioVis using the Rembrandt dataset (http://gliovis.bioinfo.cnio.es).

### Quantitative determination of the *DMRTA2* expression in human samples

Real time PCR amplifications with the primer sets: *DMRTA2 -* Hs00294890_m1; *GAPDH* - Hs02753991_g1 (Life Technologies) were performed in triplicates. The reference brain RNA was a mixture obtained from 23 normal brains (FirstChoice® Human Brain Reference RNA, Ambion, Austin, TX, USA), 5 samples were from single donors. The relative quantification of gene expression was determined with ABI PRISM 7700 using the comparative CT method.

### Preparation of DNA for bisulfide conversion and DNA methylation analyses

Tri Reagent extracted DNA was additionally digested with 600 µg/mL proteinase K and purified by phenol:chloroform extraction followed by ethanol precipitation and re-suspended in H_2_O. Next, DNA was subjected to bisulfite modification using EpiTect Kit (Qiagen) according to the manufacturer’s instructions. DNA methylation was assessed using methylation-specific PCR (MS-PCR). Primers recognizing methylated and unmethylated DNA sequences were designed using Methyl Primer Express software and sequences were as follows: DMRTA2mF: TTAAGGAGTCGTTAAGGTGC, DMRTA2mR: TCAATCAAAACGTTTAACGA, DMRTA2umF: TTTTTAAGGAGTTGTTAAGGTGT, DMRTA2umR: CCTTCAATCAAAACATTTAACAA. Commercially available methylated and unmethylated DNA (Qiagen) were used as controls. PCR products were resolved by electrophoresis on 1% agarose gel containing ethidium bromide and DNA was visualized with UV light. Densitometry analysis of the picture was performed with ImageJ.

### Immunohistochemical staining

Staining for DMRTA2 was performed on 5-μm paraffin-embedded PA and GBM tissue sections. Sections were deparaffinised at 60 °C for 2 h followed by incubation in xylene, ethanol (100, 90, 70%) and rehydration. Epitopes were retrieved by microwave boiling in pH 6.0 citrate buffer for 20 min. Endogenous peroxidase was blocked in 0.3% H_2_O_2_ in methanol for 30 min followed by blocking with 1% swine/5% horse serum. Sections were incubated overnight at 4 °C with rabbit anti-DMRTA2 antibody (Abcam cat# ab156244, dilution 1:200, in 3% horse serum), washed in PBS, incubated with a biotinylated horse anti-mouse immunoglobulin (50 pg/mL PBS), then with avidin-DH-biotinylated-HP (horseradish peroxidase) (90 μg/mL PBS) (Vector Labs., Burlingame, CA, USA) for 60 min and with 3,3′-diaminobenzidine (DAB). As a control, staining with the primary antibody was omitted. Sections were stained with hematoxylin (Sigma-Aldrich, Munich, Germany), dehydrated through ethanol, cleared in xylene and mounted. Images were obtained using Leica DM4000B microscope and DAB intensity was measured by image deconvolution using ImageJ according to modified protocol [19].

### Immunofluorescent staining

For immunofluorescent staining specimens from the Brain Tumor Tissue Bank were used. Paraffin-embedded sections were incubated in 60 °C, deparaffinised in xylene, rehydrated in ethanol (100, 90, 70%) and washed with water. Epitopes were retrieved by boiling in a pH 6.0 citrate buffer for 30 min. Endogenous peroxidase was blocked in 0.3% H_2_O_2_ in 10% methanol for 30 min followed by blocking in PBS containing 10% donkey serum in 0.1% Triton X-100 solution for 1 h and incubated overnight at 4 °C with rabbit anti-DMRTA2, and mouse anti-OLIG2 or mouse anti-CD45 or mouse anti-Smooth Muscle Actin or anti-NESTIN and sheep anti-von Willebrand Factor antibodies. All antibodies were diluted in 0.1% Triton X-100/PBS solution containing 3% of donkey serum. Next, sections were washed in PBS and incubated with corresponding secondary antibodies for 1 h at room temperature. Nuclei were counter-stained with DAPI (1µg/mL) and specimens were mounted. Images were obtained on Zeiss LSM800 Airyscan microscope. For reagent specifications, catalogue numbers and concentrations, see Supplementary Table 1. To estimate DMRTA2+ and NESTIN co-localization, images were analyzed with ImageJ software. Cells showing protein co-localization were quantified. Only cells with a visible nucleus in the DAPI staining were counted.

### Cell lines, GBM patient-derived cultures and their maintenance

Human LN18, U87-MG, LN229 and T98G glioma cells were purchased from American Type Culture Collection (ATCC, Manassas, VA, USA). Glioma cells were authenticated using the Multiplex Cell Authentication protocol (Multiplexion GmbH, Heidelberg, Germany) and tested for mycoplasma contamination once a month. Patient-derived glioma primary cultures WG1, WG4 and WG14 (GBM WHO grade 4) were generated as previously described [20, 21]. Normal human astrocytes (NHA) were purchased from Lonza (Walkersville, MD, USA) and cultured as previously described [22]. Human Umbilical Vein Endothelial Cells (HUVEC) were purchased from Gibco (Angiogenesis Starter Kit Cat. No. A14609-01, Life Technologies, Rockville, MD, USA) and cultured according to the manufacturer’s protocol in Medium 200 supplemented with Large Vessel Endothelial Supplement (LVES).

### Glioma sphere cultures

For sphere formation, LN18 glioma cells were cultured as described [21]. Briefly, cells were seeded at a low density (1500 cells/cm^2^) on non-adherent plates and cultured in DMEM/F-12 GlutaMAX™ supplemented with 2% B27, 20 ng/mL rhuman bFGF, 20 ng/mL rhuman EGF, 0.0002% heparin and antibiotics. Cells were fed every 3 days by adding 1 mL of the fresh medium. After 7 days of culture, the pictures of the spheres were taken using light microscope and spheres above 100 µm in diameter were counted. Then, the spheres were collected by centrifugation at 1200 rpm at 4 °C for total RNA isolation and protein isolation for Western blotting. WG4 and WG14 spheres were obtained as described [20].

### Preparation of protein extracts and Western blot analysis

Cells were lysed in the buffer containing phosphatase and protease inhibitors (20 mM Tris HCl, pH 6.8, 137 mM sodium chloride, 25 mM β-glycerophosphate, 2 mM sodium pyrophosphate, 2 mM EDTA, 1 mM sodium orthovanadate, 1% Triton X-100, 10% glycerol, 5 µg/mL leupeptin, 5 µg/mL aprotinin, 2 mM benzamidine, 0.5 mM DTT, 1 mM PMSF). The protein concentration was determined with the Pierce BCA Protein Assay Kit (Thermo Scientific). Protein extracts were separated on SDS-PAGE before electrophoretic transfer onto a nitrocellulose membrane (Amersham Biosciences, Germany) as described [23]. After blocking with 5% non-fat milk in TBS-T (Tris-buffered saline pH 7.6/0.15% Tween 20) the membranes were incubated with primary antibody recognizing DMRTA2 (Abcam #ab156244) diluted in a TBS-T overnight at 4 °C and then with horseradish peroxidase-conjugated anti-rabbit IgG (#PI-1000, Vector Laboratories) for one hour at RT. Immunocomplexes were visualized by using SuperSignal West Pico PLUS Chemiluminescent Substrate (Thermofisher Scientific). The membranes were stripped and re-probed with horseradish peroxidase-conjugated anti-β-Actin antibody (Sigma-Aldrich) to verify total protein loading.

### Immunofluorescent staining of cells

LN18 spheres were collected by cytospin. LN18 cells were grown on coverslips at the density of 2 × 10^5^/well in 24 well plates. Cells were fixed with 4% paraformaldehyde, postfixed with 100% methanol at −20 °C and blocked with 5% donkey serum, 1% BSA and 0.3% Triton X-100 for 1 h at room temperature. Primary antibody to DMRTA2 (Abcam cat# ab156244) was diluted 1:200 in PBS 1% BSA, 0.3% Triton X-100 containing 3% donkey serum and incubated with cells at 4 °C overnight. The cells were then incubated with anti-rabbit Alexa-555-conjugated secondary antibody (Invitrogen). After several washes with PBS containing 0.1% BSA, 0.3% Triton-X100, the cells were counterstained with 1 μg/mL DAPI (Sigma). The coverslips were dried, mounted on slides and visualized by fluorescence microscopy.

### Knock-down of DMRTA2 in human LN18 glioma adherent cells

LN18 glioma cells were seeded on 96-well microplates (at the density of 4 ×10^3^/well) for MTT metabolism and BrdU incorporation assays and 6-well plates (at the density of 2.25 × 10^5^/well) for total RNA and protein isolation and left overnight to attach. The next day, the culture medium was removed and cells were transfected with 25 nM control or DMRTA2-specific siRNA (Dharmacon) using Viromer® BLUE transfection reagent (Lipocalyx GmbH, Halle, Germany). The culture medium was replaced after 4 h. The cells were assessed for cell viability and proliferation or collected for total RNA and protein isolation for Western Blot or harvested and seeded at a low density for the sphere formation assay. The efficiency of gene knock-down was determined by qPCR and Western blot analysis.

### Knock-down of DMRTA2 in glioma-derived spheres

LN18 spheres cultured for 10 days or WG14 spheres cultured for 21 days were collected by centrifugation, washed with PBS and dissociated to single-cell suspension using TrypLe Express (Thermo Fisher Scientific). 1 × 10^5^ cells were transfected with 50 nM of control or DMRTA2-specific siRNA (Dharmacon) in 20 μl of SF buffer (SF Cell Line 4D-Nucleofector ^TM^ X Kit) using 4D-Nucleofector system (Lonza). After transfection, cells were immediately resuspended in a fresh medium for spheres without antibiotics, plated on 60 mm plates for cell suspension and incubated for 24 h.

### Tube formation assay

Pre-chilled 48-well culture plates were coated with Geltrex® LDEV-Free Reduced Growth Factor Basement Membrane Matrix (Angiogenesis Starter Kit # A14609-01, Gibco, Life Technologies) and incubated at 37 °C for 30 min. Meanwhile, the LN18 or WG14 spheres were dissociated to single-cell suspension using TrypLe Express (Thermo Fisher Scientific) and cells were counted. Then, 5 × 10^4^ cells were stained with Vybrant™ DiI Cell-Labeling Solution (Thermo Fisher Scientific) following manufacturer instructions, washed with PBS and re-suspended in Medium 200 without a supplement. Human umbilical vein endothelial cells (HUVEC) were trypsinized, washed with PBS and counted. 7 × 10^4^ HUVEC cells in 200 μL and 1 × 10^4^ LN18 cells from spheres in 100 μL Medium 200 without a supplement were plated on 48-well plate previously coated with Geltrex® LDEV-Free Reduced Growth Factor Basement Membrane Matrix. Large Vessel Endothelial Supplement (LVES) was added as a control. The cells were visualized 16 h after seeding with fluorescent microscopy for endothelial tube formation and pictures were taken for angiogenesis analysis with ImageJ.

### scRNA-seq data analysis

Published single-cell RNA-seq count matrices for GBM patients were obtained [24]. Data analysis was performed in R using Seurat v4 [25]. All samples (*N* = 14, mean number of cells per sample = 3829) were first filtered to remove dying cells (> 8% Unique Molecular Identifiers to mitochondrial genes, <5,5% UMI to ribosomal genes) and low complexity cells (<200 genes expressed per cell). Subsequently, *MALAT1*, ribosomal genes and genes with less than 20 counts have been discarded according to 10X Genomics recommendations. Samples were joined together using the *“merge”* function from the Seurat package. Next, raw counts were normalized to log_2_(RPKM+1). Expression levels were scaled for each gene by dividing the centered feature expression levels by their standard deviations. Multiplets (two or more cells with the same 10X barcode) were identified with DoubletFinder [26] and next discarded from the analysis. All major cell populations (with the exception of neural cells) have been identified: myeloid cells, lymphoid cells, oligodendrocytes, astrocytes, pericytes, and endothelial cells (markers used for identification are in Supplemental Table 2). The SOX2^+^ clusters were regarded as neoplastic and further analyzed.

### Statistical analysis

All biological experiments were performed on 3–4 independent cell passages. Results are expressed as means ± standard deviation (SD). P-values were calculated using two-tailed *t* test or one-way ANOVA followed by appropriate post-hoc test using GraphPad Prism v6 (GraphPad Software, USA). Differences were considered statistically significant for *p* values < 0.05. Moreover, we calculated the effect size (Hedge’s ‘g’ between the groups) [27]. A commonly used interpretation for effect size is as follows: small (0.2), medium (0.5) and large (0.8); however, these values are arbitrary and should not be considered rigidly [27, 28].

## Results

### DMRTA2 is highly expressed in human glioblastoma and predicts poor survival

**Fig. 1:**
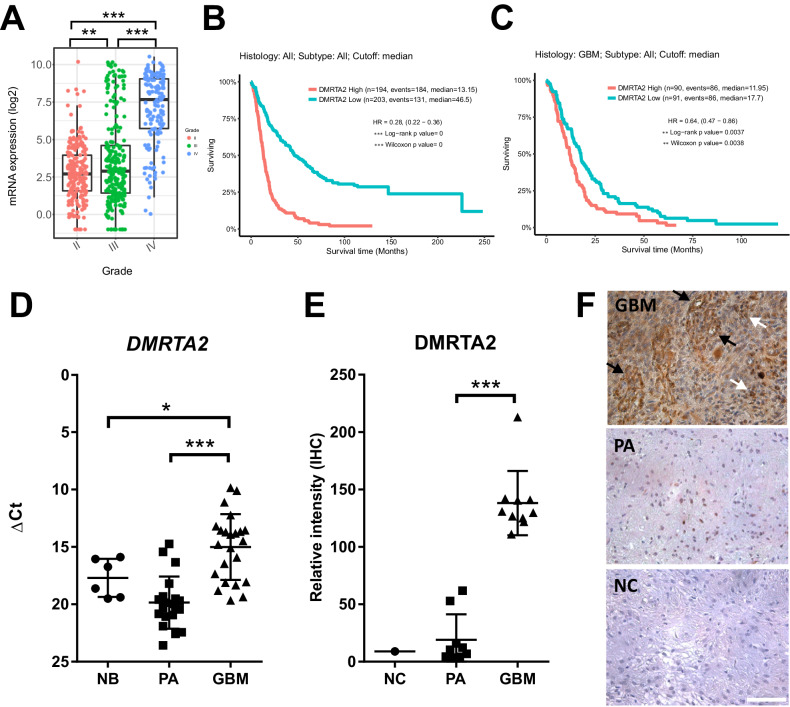
*DMRTA*2 is overexpressed in high grade gliomas, and its high expression negatively correlates with glioma patient survival. **A** Analysis of *DMRTA2* mRNA expression in the TCGA data set using GlioVis. **B**, **C** Kaplan–Meier survival plots of all gliomas (**B**), and glioblastoma (**C**), patients. Patients were assigned to groups according to *DMRTA2* expression levels. **D** Quantification of *DMRTA2* mRNA levels in pilocytic gliomas (20 patients) and glioblastomas (23 patients) determined by qRT-PCR. **E** Quantification of immunohistochemistry staining for DMRTA2 in high and low-grade gliomas. **F** Representative images of immunohistochemical staining for DMRTA2 in high and low grade gliomas, black arrows point to positive staining around vessels, white arrows mark small cells with high proliferation potential, scale bar—100 µm. GBM glioblastoma, PA pilocytic astrocytoma, NB normal brain, NC negative control; *p < 0.05, **p < 0.01, ***p < 0.001.

To explore *DMRTA2* expression in brain tumors we took advantage of GlioVis, the open access web application for data visualization and analysis. Using RNA-sequencing data from the TCGA dataset, the levels of *DMRTA2* mRNA were evaluated in a large group of gliomas of different WHO grades (G2-3-4). The *DMRTA2* expression increased in tumors with higher malignancy grade and was significantly higher in GBM when compared to lower grade gliomas (Fig. 1A). In order to assess if *DMRTA2* expression affects patient outcome we used the Rembrandt (REpository of Molecular BRAin Neoplasia DaTa) database [29]. Expression of *DMRTA2* was strongly associated with patient survival in all gliomas (Fig. 1B), which is also evident among GBMs, highlighting the DMRTA2 involvement in GBM malignant phenotype (Fig. 1C). We confirmed high expression of *DMRTA2* in our cohort of GBM samples in comparison to juvenile pilocytic astrocytoma (PA) and normal brain tissue (NB) (Fig. 1D). PA is a slow growing, not diffusive tumor [30], which rarely undergoes progression to high-grade tumors [31]. Immunohistochemistry staining confirmed high expression of DMRTA2 protein in malignant gliomas compared to benign tumors (Fig. 1E, F). In GBM sections positive DMRTA2 staining was evident in two distinct cell populations: first are small, highly proliferative cells present in tumor parenchyma, and the other are cells localized around blood vessels.

### DMRTA2 positive cells exhibit certain stem-like and pericyte-like properties

**Fig. 2:**
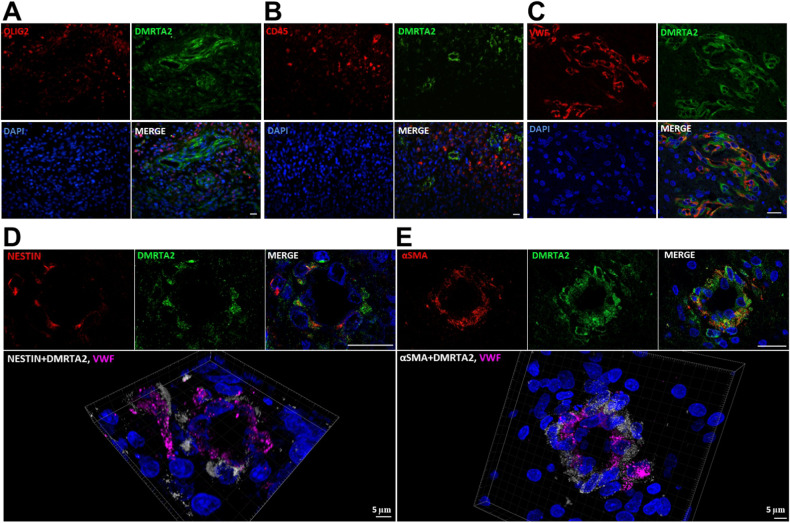
Localization of DMRTA2 positive cells in GBMs. Representative images of immunofluorescence staining for DMRTA2 and (**A**) OLIG2, (**B**) CD45, (**C**) von Willebrant factor -VWF, (**D**) NESTIN and (**E**) αSMA in glioblastoma. Nuclei visualized with DAPI staining. Scale bar −20 µm, unless indicated otherwise.

To characterize the functions of DMRTA2 positive cells, we performed the Gene Ontology (GO) biological processes analysis of glioma samples in the Rembrandt database. Tumors expressing high levels of *DMRTA2* were distinguished by the enrichment in terms such as extracellular structure/matrix organization, blood vessel morphogenesis and angiogenesis (Supplemental Fig. 1). Further, we analyzed public scRNA-seq data from GBMs [24]. To characterize the cell identity of the obtained clusters, we applied the panel of markers identifying specific cells present in the brain and analyzed their co-expression with *DMRTA2*. All major cell populations have been identified, and non-tumor cells were removed from the further analysis. The remaining clusters expressed *SOX2* and were regarded as neoplastic cells (Supplemental Fig. 2A). We analyzed these cells for the expression of neural/stem precursor cell markers (*OLIG1, OLIG2, CSPG4/NG2, NESTIN*), which are also transcription factors and lineage markers associated with glioma stem cells (GSC) [32] (Supplemental Fig. 2C), and *DMRTA2* (Supplemental Fig. 2B). *DMRTA2* expressing cells within S*OX2* expressing clusters co-expressed also oligodendrocyte (*OLIG1, OLIG2, CSPG4*) and/or neural (*NESTIN*) progenitor cell genes (Supplemental Fig. 2C, E). Considering these data and immunohistochemistry staining of DMRTA2 in GBM, we focused on determining DMRTA2 co-expression with markers of stem/progenitor cells (OLIG2 [33] and NESTIN [34]), immune cells (CD45 [35]) and blood vessels (von Willebrant factor—VWF [34]). We found that neither OLIG2 nor CD45 co-localize with DMRTA2 staining (Fig. 2A, B), but we observed co-localization of DMRTA2 with NESTIN (Fig. 2D and Supplemental Fig. 3). Moreover, we noticed DMRTA2 positive cells in a close proximity to those positive for an endothelial marker—VWF (Fig. 2C). Many of DMRTA2^+^NESTIN^+^ cells were found around VWF^+^ endothelial cells. Vascular pericytes stretch around endothelial cells (ECs) and are functionally associated with regulating vessel stabilization, vessel diameter and EC proliferation. Some pericyte cells, which participate in angiogenesis, express NESTIN [36]. Therefore, we performed staining for another pericyte marker—α-Smooth Muscle Actin (αSMA), together with DMRTA2 and VWF. αSMA was found in pericytes supporting vessel formation in GBM [37]. We detected αSMA^+^ cells among DMRTA2^+^ cells (Fig. 2E), and these cells were localized next to VWF^+^ cells forming vessels. This suggests that DMRTA2^+^ cells contain a subpopulation of cells which acquire pericyte-specific features (Fig. 2E). The analysis of scRNA-seq data demonstrated a subset of *DMRTA2* expressing cells having pericyte markers: out of which *ACTA2 (*coding for αSMA*)* and/or *SIPR3* (coding for Sphingosine-1-Phosphate Receptor 3) and/or *MCAM (*coding for Melanoma Cell Adhesion Molecule, known as cluster of differentiation 146) were most frequently expressed (Supplemental Fig. 2D, F).

### DMRTA2 is overexpressed in glioma sphere-forming cells and supports their self-renewal

**Fig. 3:**
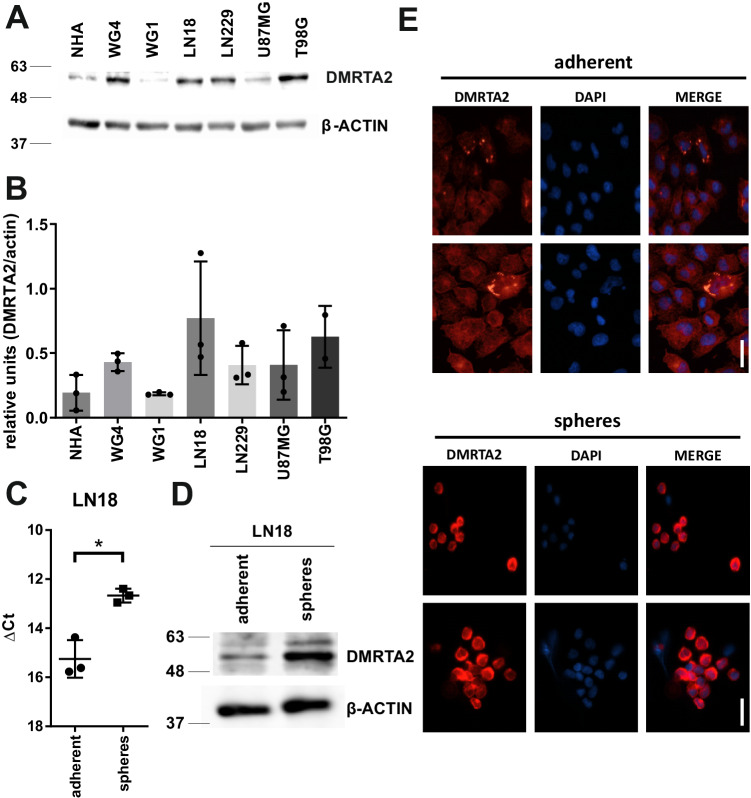
DMRTA2 expression is upregulated in human glioma cells, in particular in sphere cultures enriched in glioma stem cells. **A** Western blot showing DMRTA2 protein levels in established glioma cells (LN18, U87MG, T98G, LN229), patient-derived (WG4, WG1) glioma cell cultures and normal human astrocytes (NHA). **B** Densitometry analysis of Western blot for DMRTA2 protein expression in normal human astrocytes (NHA), established (LN18, U87MG, T98G, LN229) and patient-derived (WG4, WG1) glioma cell lines (2–3 biological replicates for each cell line). **C** Quantification of *DMRTA2* expression in LN18 adherent cells and LN18-derived spheres by qRT-PCR (*n* = 3 biological replicates). *p < 0.05. **D** Representative immunoblot showing the DMRTA2 protein levels in LN18 cells and LN18-derived spheres. **E** Immunofluorescence for DMRTA2 in LN18 adherent cells and LN18-derived spheres (*n* = 3 biological replicates). Nuclei visualized with DAPI, scale bar—50 µm. Of note is uniform, nuclear expression of DMRTA2 in most LN18 glioma sphere-forming cells, while in adherent cells DMRTA2 is visible in a cytoplasm and rare cells.

We analyzed DMRTA2 expression in the panel of primary and established glioma cell lines. The highest level of DMRTA2 protein was detected in two established cell lines: LN18 and T98G and primary GBM-derived WG4 cell cultures (Fig. 3A, B and Supplemental Fig. 4A). LN18 and WG4 cells have been previously described as forming spheres enriched in cells with cancer stem cell properties when cultured under proper conditions [21]. Increased DMRTA2 expression was indeed detected in spheres at both mRNA and protein level, when compared with adherent cells (Fig. 3C–E and Supplemental Figs. 4B, 5). These results are in line with scRNA-seq and immunofluorescence results, and implicate DMRTA2 in GSC functions.

**Fig. 4:**
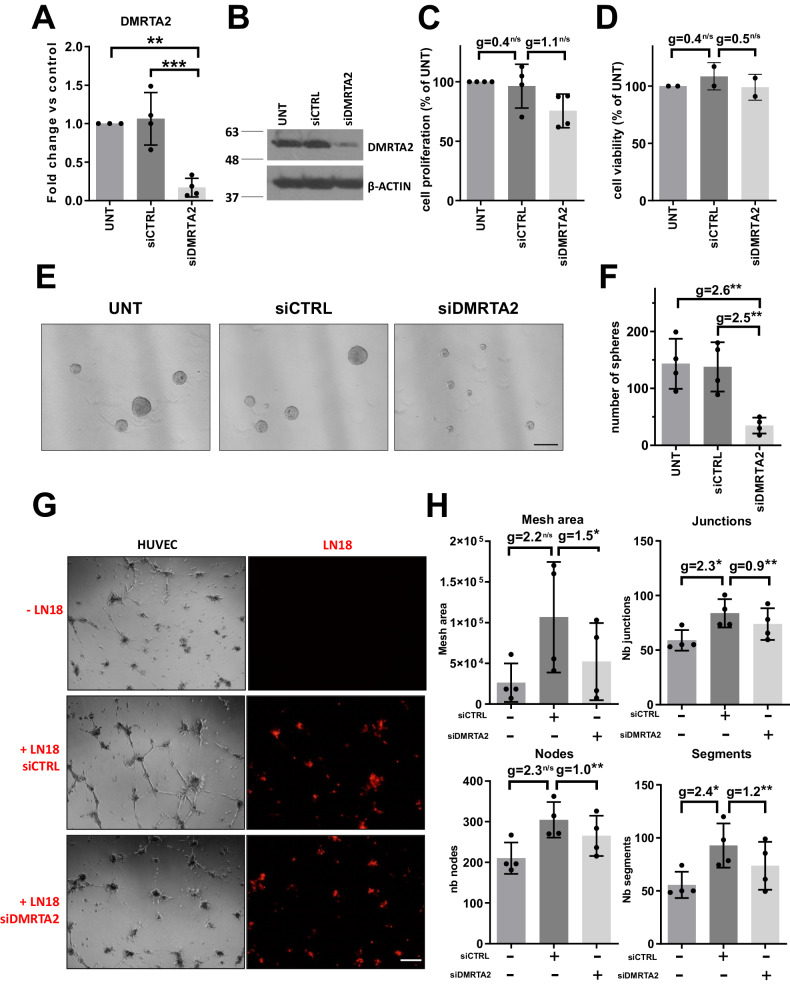
DMRTA2 is indispensable for LN18 sphere formation and glioma stem cells driven support of angiogenesis. **A** The efficacy of DMRTA2 knock-down was evaluated by qRT-PCR 48 h after siRNA transfection (*n* = 4 biological replicates). **B** Western blot showing reduction of the DMRTA2 protein level in LN18 cells after DMRTA2 knock-down. **C** Cell proliferation of LN18 cells decreased in DMRTA2-depleted cells as verified with BrdU incorporation test (*n* = 4 biological replicates in triplicates). **D** Viability of LN18 cells was not affected by DMRTA2 knock-down as evaluated using MTT metabolism test (*n* = 2 biological replicates in triplicates). **E** Sphere formation capacities decrease in LN18 cells depleted of DMRTA2. Representative photos of LN18-derived spheres untreated, or transfected with siCTRL or siDMRTA2 and cultured for 7 days under sphere-forming conditions, scale bar—200 µm. **F** Number of spheres formed by LN18 cells was reduced after DMRTA2 knock-down (*n* = 4 biological replicates). **G** Representative photos of vascular nets formed by HUVEC alone or in co-culture with control LN18 or DMRTA2 depleted cells, scale bar—200 µm. **H** Quantification of selected properties of the vascular net formed by HUVEC alone or in co-culture with control or DMRTA2 depleted LN18 cells (*n* = 4 biological replicates). UNT—control untreated cells; *p < 0.05; **p < 0.01; ***p < 0.001; Hedge’s ‘g’ stands for effect size.

To investigate the role of DMRTA2 in glioma stem-like cells, we efficiently silenced DMRTA2 expression in LN18 cells (Fig. 4A, B and Supplemental Fig. 6). Loss of DMRTA2 expression impaired proliferation but did not affect viability of LN18 cells (Fig. 4C, D, respectively). Furthermore, DMRTA2-depleted cells had reduced potential to form spheres in vitro (Fig. 4E, F). The corresponding effect size of sphere number reduction in DMRTA2-depleted cells was high (*g* = 2.5) emphasizing its role in GSC self-renewal.

### Loss of DMRTA2 disrupts vascular net created in vitro by endothelial cells and stabilized by glioma stem-like cells

Immunofluorescent staining revealed the presence of the DMRTA2 protein in the cells surrounding blood vessels. Most of these cells were also positive for αSMA, a pericyte marker. Pericytes stabilize and monitor the maturation of endothelial cells by direct communication with those cells as well as through paracrine signaling [38]. Some reports suggested glioblastoma stem cells may acquire a pericyte-like phenotype and this way contribute to tumor neovascularization [37]. We detected DMRTA2 positive cells with αSMA expression around endothelial cells (Fig. 2E). To study a potential role of DMRTA2 in tumor neovascularization, we employed an in vitro tube formation assay which measures the ability of endothelial cells, plated at sub-confluent densities to form capillary-like structures (tubes) provided with the appropriate extracellular matrix support [39]. Tube formation occurs quickly with endothelial cells aligning on a matrix within 1 h and is completed within 12–20 h following endothelial cell plating. After that time, the in vitro formed net starts to collapse without further support from angiogenic factors [39]. We performed a tube formation assay with human HUVEC cells in the presence of LN18 or WG14 cells grown as spheres. We selected the WG14 cell cultures due to the high expression of *DMRTA2* (RNA-seq data in [20]) and the ability to form spheres. HUVEC cells plated on matrigel formed tubules within 12 h and at 16 h we observed disruption of the vascular net (Supplemental Figure 7). When HUVEC cells were plated in co-cultures with LN18 or WG14 cells derived from spheres, we observed stabilization of the formed vessels and the vascular net was still present at 16 h after plating (Fig. 4G, Supplemental Fig. 7, 8A). When HUVEC cells were co-cultured with sphere-derived, DMRTA2 depleted LN18 or WG14 cells (Fig. 4G, Supplemental Fig. 8A), we observed the decrease of mesh area, and the reduced numbers of nodes, junctions and segments in the vascular network formed by HUVEC (Fig. 4G, H and Supplementary Fig. 8A, B). Moreover, the corresponding effect size was high in each performed comparison between groups of interest, underlining the role of DMRTA2 in sustaining the vascular network stability.

### Increased expression of *DMRTA2* in high-grade gliomas is associated with DNA methylation

**Fig. 5:**
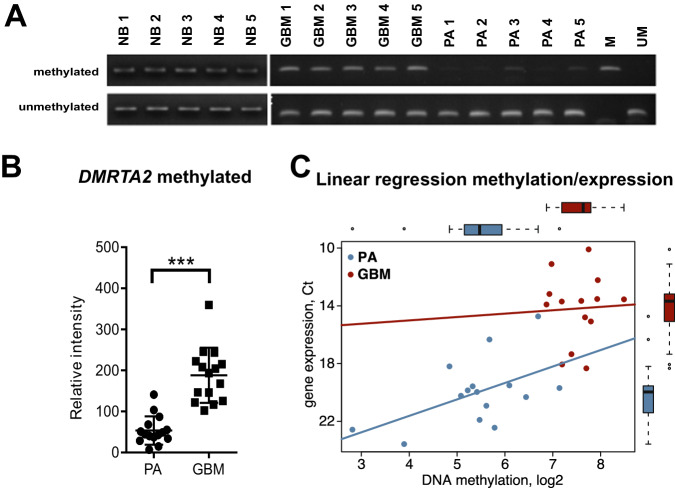
Methylation of *DMRTA2* correlates with its expression in gliomas. **A** Representative results of MS-PCR for the CpG sites located in the 3′ UTR of the *DMRTA2* gene. **B** Densitometry analysis of MS-PCR results relative to methylated control DNA (15 GBM and 15 PA samples). **C** Analysis of correlation of DNA methylation and *DMRTA2* gene expression in high (14 samples) and low (15 samples) grade gliomas—*p* < 0.0001, *r* = −0.7150. GBM glioblastoma, PA pilocytic astrocytoma, NB normal brain, M methylated control, UM unmethylated control.

Epigenetic changes are considered to be among the earliest and most comprehensive genomic aberrations occurring during carcinogenesis [40]. The contribution of epigenetic mechanisms to glioblastoma pathology has been broadly studied and numerous alterations in DNA methylation patterns were detected [41]. To decipher a potential mechanism responsible for aberrant *DMRTA2* expression in GBM, we sought to determine if DNA methylation alterations are responsible for the upregulation of *DMRTA2* expression. We analyzed the *DMRTA2* coding sequence for the presence of CpG island using EMBOSS Cpgplot. The whole *DMRTA2* coding sequence appeared to be enriched for CpG dinucleotides. We took advantage of the DNA methylation data from Stepniak et al., [42] and looked at the DNA methylation within the *DMRTA2* gene in GBM and PA. We observed differences in DNA methylation in the gene body and at the 3′ UTR of *DMRTA2* [42]. Using methyl-specific PCR we found both methylated and unmethylated *DMRTA2* sequences in DNA isolated from GBM, whereas no or very low amplification of methylated sequences was detected in PA samples (Fig. 5A, B). Furthermore, both methylated and unmethylated sequences were detected in DNA isolated from non-cancerous brain tissues (5 samples) (Fig. 5A); however, no or very low levels of *DMRTA2* transcripts were found in contrast to GBM highly expressing *DMRTA2* (Fig. 1D). Correlation analysis revealed a positive correlation between the DNA methylation level and *DMRTA2* expression in the analyzed cohort of samples (Fig. 5C). These results suggest that DNA methylation may contribute to *DMRTA2* expression in GBM.

## Discussion

The transcription factor DMRTA2 is involved in gonadal differentiation and CNS development taking part in determining cell fate decisions. The potential role of DMRTA2 as a prognostic marker was indicated in several non-CNS cancers as this gene appeared deregulated in gene expression [43, 44] or DNA methylation-based signatures [45, 46]. Tumor cell origin studies indicated glioblastoma originates from neural stem/progenitor cells. Therefore, any factors controlling cell lineage fate or differentiation are of particular interest, but the role of DMRTA2 in gliomagenesis has been unknown.

In this study, we determined the expression of *DMRTA2* in human gliomas of different WHO grades, primary GBM-derived cultures and established glioma cell lines, and we found upregulation of *DMRTA2* in high-grade gliomas. The DMRTA2 protein accumulated around blood vessels and in small cells with high proliferative potential in GBM sections. Such cells are consistent with primitive, quiescent stem cells residing in adult tissues suggesting a correlation between small cell sizes with the stemness [47]. ScRNA-seq data revealed that *DMRTA2* expressing cells exhibited high expression of markers characteristic for stem and progenitor cells: *SOX2, OLIG1, OLIG2, Nestin, CSPG4* (*NG2*) (Supplemental Fig. 2) supporting a link between DMRTA2 expression and the GSC phenotype. Furthermore, expression of DMRTA2 was increased in cultured glioma cells compared with normal human astrocytes. GBM-derived spheres enriched for cancer stem cells had higher DMRTA2 expression at mRNA and protein levels than adherent cells. Knock-down of DMRTA2 in glioma cells did not affect cell viability but affected proliferation and the GCS self-renewal. These results suggest a role of DMRTA2 in stemness maintenance and dysregulation of the differentiation program. DMRTA2 has been demonstrated as a factor in maintaining NPC in the cell cycle and preventing differentiation during brain development [16]. The presence of GCS is an important hallmark of GBM [32, 48].

We found DMRTA2-positive cells expressing pericyte markers around blood vessels in GBMs. Vascular pericytes play critical roles in supporting vascular structure and function, maintaining the blood-brain barrier, facilitating vessel maturation, and initiation of vessel sprouting [37]. However, the pericyte-endothelial cells interactions change substantially in tumors [49, 50]. The tumor vasculature frequently exhibits structural and functional abnormalities with irregular pericytes on endothelial tubules. In vivo cell lineage tracing demonstrated that GSC may generate vascular pericytes [37]. Our results suggest that DMRTA2 could be a marker of such GSC-derived pericytes as these cells co-express pericyte-specific markers as indicated by scRNA-seq data and immunofluorescence staining. Of particular interest is the presence of NESTIN as NESTIN^+^ (but not NESTIN^-^) pericytes were recruited to the blood vessels during tumor angiogenesis in the murine intracerebral glioblastoma [36]. We demonstrate the supportive role of cells derived from LN18 and WG14 spheres in stabilization of the vascular net created by endothelial cells in vitro. This support is lost when DMRTA2 is depleted in GSCs. The blood-tumor barrier (BTB) is a major obstacle to drug delivery to GBM but could be also a new attractive target [51]. Targeting GSC-derived pericytes may potentially enhance drug delivery, improving GBM therapy.

We explored DNA methylation as a potential mechanism through which *DMRTA2* expression could be deregulated in GBMs. The *DMRTA2* methylation level was described as a poor prognosis factor in Clear Cell Renal Cell Carcinoma [45] and as prognostic factor in bladder cancer [46]. However, the analysis of DNA methylation within CpG islands in the 3’ UTR of the *DMRTA2* gene showed that despite a similar methylation pattern of the *DMRTA2* in normal brain and GBM, the gene was expressed only in malignant samples. On the other hand, the *DMRTA2* gene was not methylated at the tested sites in PA samples and the gene was not expressed. DNA methylation in different genomic regions may exert a different influence on gene activity, and DNA methylation of the gene body was associated with a higher level of gene expression in dividing cells [52, 53]. In slowly dividing and non-dividing cells such as the cells of the brain, gene body methylation was not associated with increased gene expression [53, 54]. These data are in agreement with our findings of high expression of the *DMRTA2* gene in proliferating, malignant cells and not detectable expression in normal brain tissue. However, the presence of DNA methylation in the 3’ UTR of *DMRTA2* may also affect the binding of repressors and this effect could result in high expression of *DMRTA2* in GBMs. Further studies to elucidate the role of DNA methylation in DMRTA2 expression regulation in glioblastoma and its implication in glioma angiogenesis are necessary, as some DNA methylation inhibitors markedly decrease vessel formation in different tumor in vitro and in vivo models [55].

Altogether, our findings point to a role of DMRTA2, highly overexpressed in GBM, in glioma pathogenesis. Differential DMRTA2 expression in low and high-grade gliomas and in cultured glioma spheres enriched in GSC suggests dysregulation of the differentiation program. Moreover, the involvement of DMRTA2 in glioblastoma neovascularization opens a new possibility of targeting the brain-tumor barrier, which may facilitate drug delivery to GBM to effectively block tumor progression and improve therapy.

### Supplementary information

Supplemental information

Original Data File

aj-checklist_CDDIS-23-3901-T

## Data Availability

The datasets analyzed during the current study are available in the GlioVis web application for data visualization and analysis, http://gliovis.bioinfo.cnio.es/. ScRNA-seq data are avialable from the authors of the original study on special request.
