# Supplementary material for: DMRTA2 supports glioma stem-cell mediated neovascularization in glioblastoma
Source: Cell Death Dis. 2024 Mar 20;15(3):228. doi: 10.1038/s41419-024-06603-y (PMC10954651; doi:10.1038/s41419-024-06603-y)
Supplement: Supplementary file 1 — Supplemental information [file 41419_2024_6603_MOESM1_ESM.pdf]

Supplemental information

Supplemental Table 1. List of antibodies used in immunochemical and immunofluorescent staining.

Reagent	Manufacturer	Cat. number	Clone	Fluorophore	Dilution
anti-DMRTA2, pAb	Abcam	ab156244	-	-	1:100
anti- OLIG2	Millipore	MABN50	-	-	1:100
anti-CD45 (HLA-DP,DQ,DR)	Dako	M0775	CR3/43		1:200
anti-SMA	Santa-Cruz	sc-53142	B4	-	1:100
anti-NESTIN	Cell Signaling	33475	-	-	1:200
anti-von Willebrand Factor, pAb	Abcam	ab11713	-	-	1:1000
anti-rabbit AF488	Invitrogen	A-21206	-	AF488	1:2000
anti-mouse AF555	Invitrogen	A-31570	-	AF555	1:2000
anti-sheep AF647	Abcam	ab150179	-	AF647	1:2000

Supplemental Table 2. List of literature-based markers used to create a marker panel for characterization of cell identity of obtained clusters.

Gene name	Target group	Reference
CD14	myeloid cells	(1)
CD3	lymphoid cells	(1)

OLIG1	oligodendrocytes	(2)
OLIG2	oligodendrocytes	(2)
GFAP	astrocytes	(3)
TUBB3	neurons	(4)
RBFOX3	neurons	(4)
PDGFRB	pericytes	(5)
ACTA2	pericytes	(5)
PECAM	endothelial cells	(6)
ENG	endothelial cells	(7)
PTPRC	Microglia/macrophages	(8)
SOX2	tumor cells	(1)

1. Yuan J, Levitin HM, Frattini V, Bush EC, Boyett DM, Samanamud J, et al. Single-cell transcriptome analysis of lineage diversity in high-grade glioma. Genome Med. 2018;10(1):57.
2. Ohnishi A, Sawa H, Tsuda M, Sawamura Y, Itoh T, Iwasaki Y, et al. Expression of the oligodendroglial lineage-associated markers Olig1 and Olig2 in different types of human gliomas. J Neuropathol Exp Neurol. 2003;62(10):1052-9.
3. Furnari FB, Fenton T, Bachoo RM, Mukasa A, Stommel JM, Stegh A, et al. Malignant astrocytic glioma: genetics, biology, and paths to treatment. Genes Dev. 2007;21(21):2683-710.
4. Ju ZH, Liang X, Ren YY, Shu LW, Yan YH, Cui X. Neurons derived from human-induced pluripotent stem cells express mu and kappa opioid receptors. Neural Regen Res. 2021;16(4):653-8.
5. Cheng L, Huang Z, Zhou W, Wu Q, Donnola S, Liu JK, et al. Glioblastoma stem cells generate vascular pericytes to support vessel function and tumor growth. Cell. 2013;153(1):139-52.
6. Schley M, Ständer S, Kerner J, Vajkoczy P, Schüpfer G, Dusch M, et al. Predominant CB2 receptor expression in endothelial cells of glioblastoma in humans. Brain Res Bull. 2009;79(5):333-7.

7. Serrano-Garrido O, Peris-Torres C, Redondo-García S, Asenjo HG, Plaza-Calonge MDC, Fernandez-Luna JL, et al. ADAMTS1 Supports Endothelial Plasticity of Glioblastoma Cells with Relevance for Glioma Progression. Biomolecules. 2020;11(1).
8. Ochocka N, Segit P, Walentynowicz KA, Wojnicki K, Cyranowski S, Swatler J, et al. Single-cell RNA sequencing reveals functional heterogeneity of glioma-associated brain macrophages. Nat Commun. 2021;12(1):1151.

Rembrandt database, GO analysis, ALL glioma

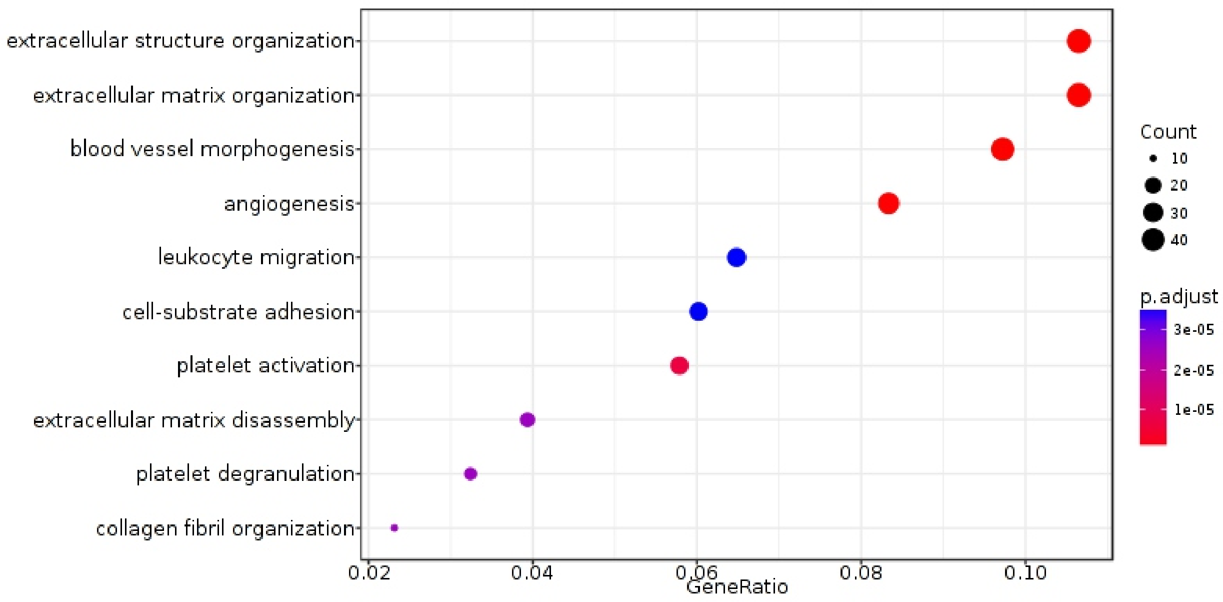

Supplemental figure 1. Gene Ontology (biological processes) enrichment analysis of glioma samples according to DMRTA2 expression performed using GlioVis.

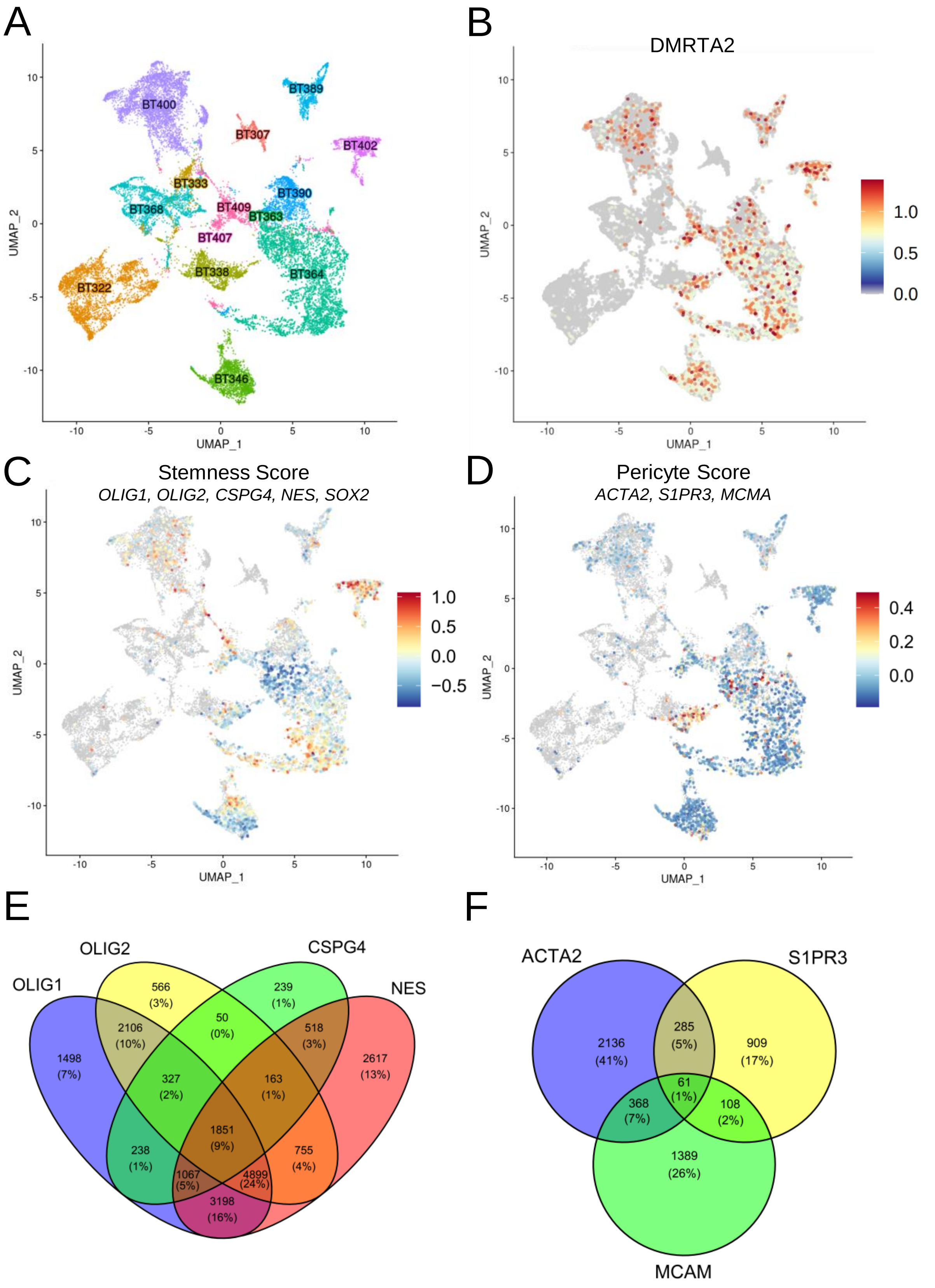

A
B
DMRTA2
C
Stemness Score
OLIG1, OLIG2, CSPG4, NES, SOX2
D
Pericyte Score
ACTA2, S1PR3, MCMA
UMAP_1
UMAP_2
BT400
BT389
BT307
BT402
BT333
BT390
BT368
BT409
BT363
BT407
BT338
BT364
BT322
BT346
E
OLIG2
CSPG4
OLIG1
NES
566 (3%)
239 (1%)
50 (0%)
2106 (10%)
518 (3%)
1498 (7%)
2617 (13%)
327 (2%)
163 (1%)
1851 (9%)
238 (1%)
755 (4%)
1067 (5%)
4899 (24%)
3198 (16%)
F
ACTA2
S1PR3
MCAM
2136 (41%)
285 (5%)
909 (17%)
61 (1%)
368 (7%)
108 (2%)
1389 (26%)

Supplemental figure 2. The scRNA-seq analysis of expression of DMRTA2 and stem cell markers in glioblastoma in publicly available datasets (Couturier et al, 2020) 24. (A) Umap of 23280 cells from GBM patients colored by patient. (B) Umap representing expression of DMRTA2. Cells without DMRTA2 expression are colored gray. (C,D) UMAPs of stemness and pericytes scores in DMRTA2+ cells. (E) Venn diagram showing percentage of DMRTA2+ cells co-expressing selected stem/progenitor cell markers. (F) Venn diagram showing percentage of SOX2+/DMRTA2+ cells co-expressing selected pericyte markers.

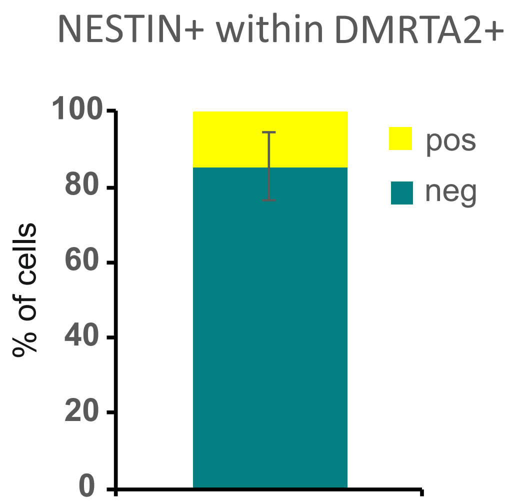

Supplemental figure 3. Quantification of DMRTA2 and NESTIN coexpression in GBM tissue (n=3).

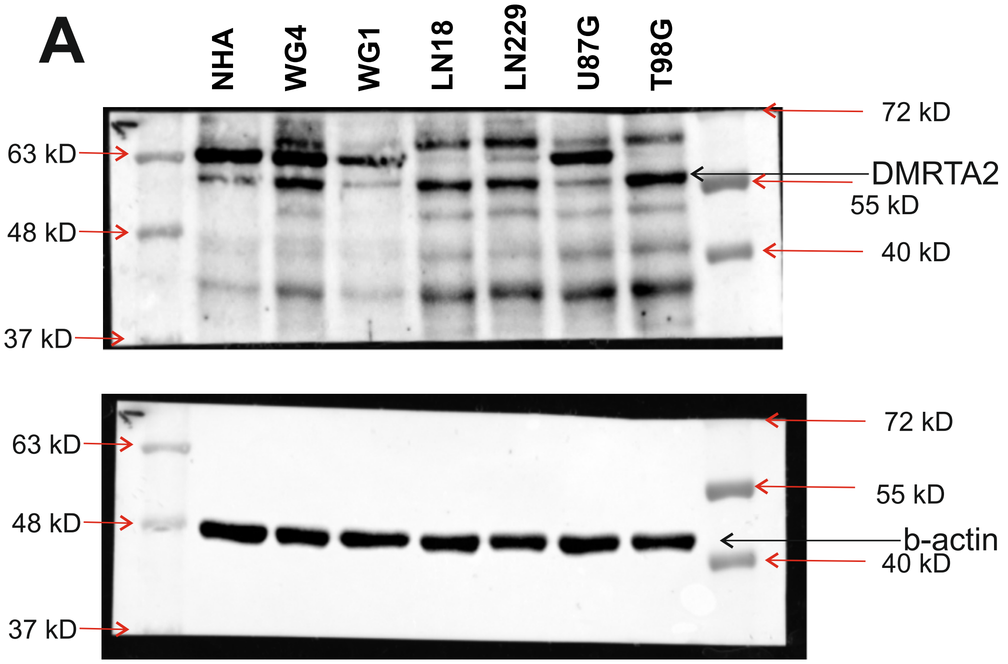

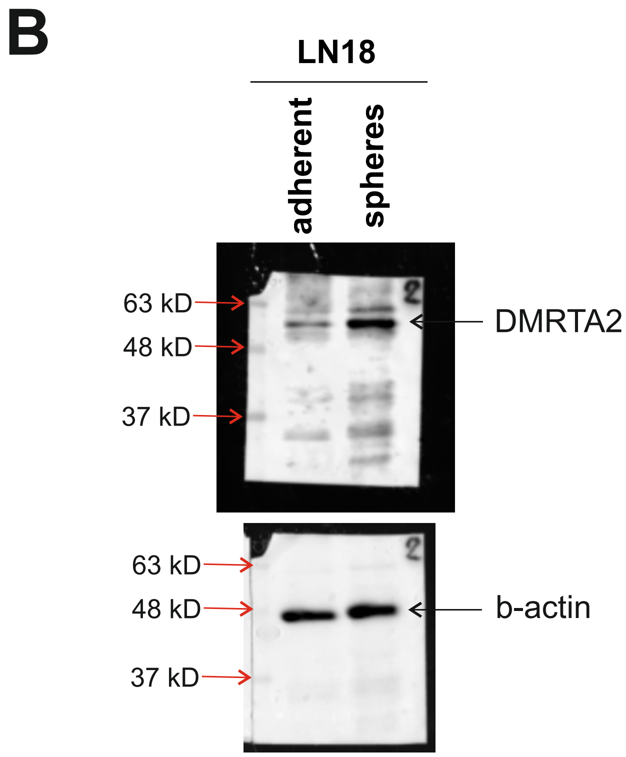

Supplemental figure 4. Original Western blots to A) Figure 3A; B) Figure 3D

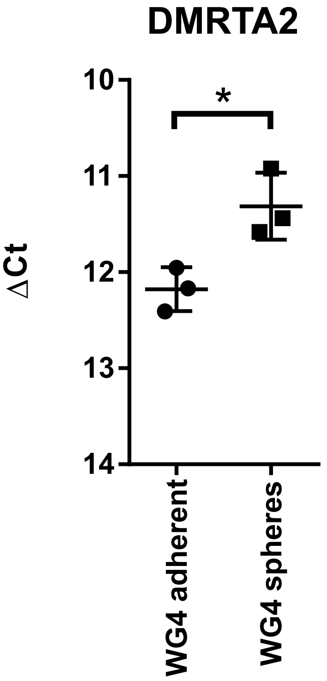

Supplemental figure 5. Quantification of DMRTA2 expression in WG4 adherent cells and WG4-derived spheres. * - p< 0.05

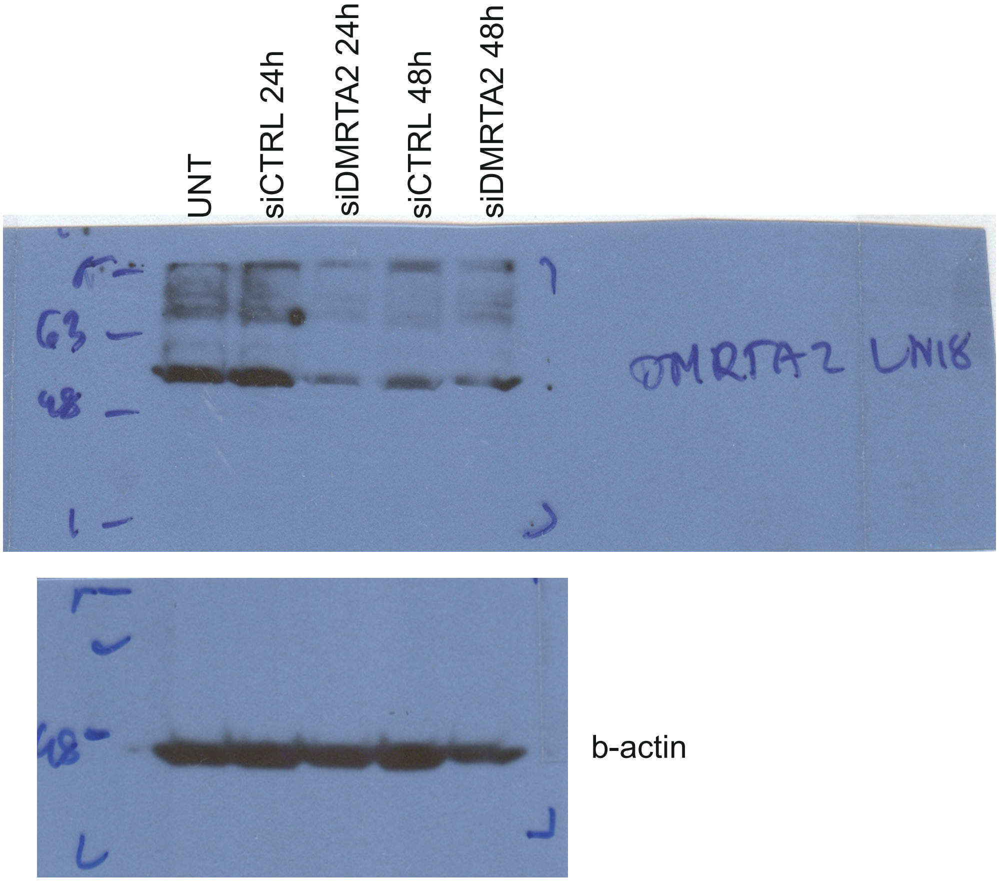

Supplemental figure 6. Original Western blots to Figure 4B.

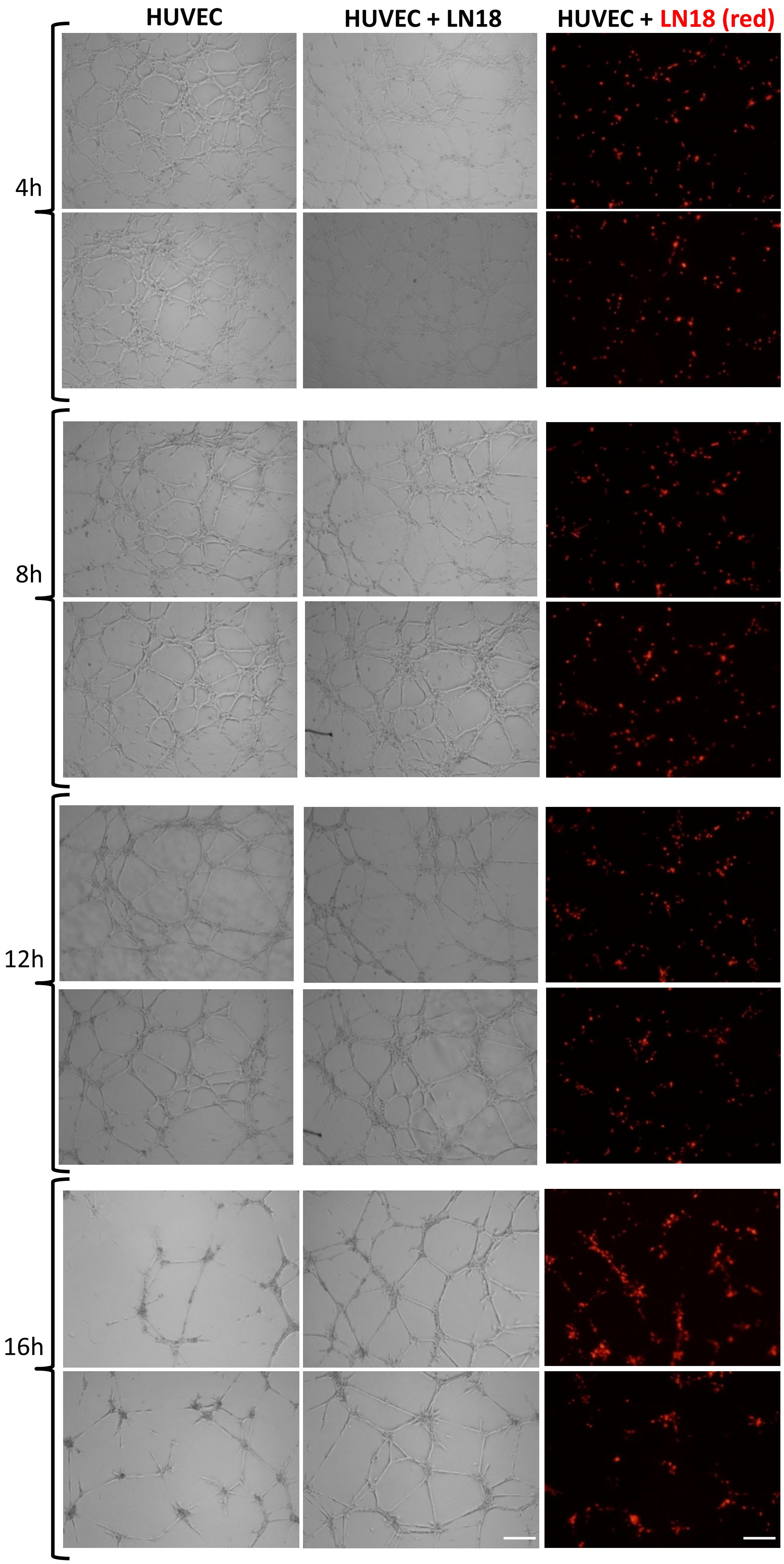

Supplemental figure 7. Results of tube forming assay by HUVEC. Representative photos of the vascular net formed by HUVEC cultured alone or at different times after co-culture with LN18 cells, scale bar – 200 μm.

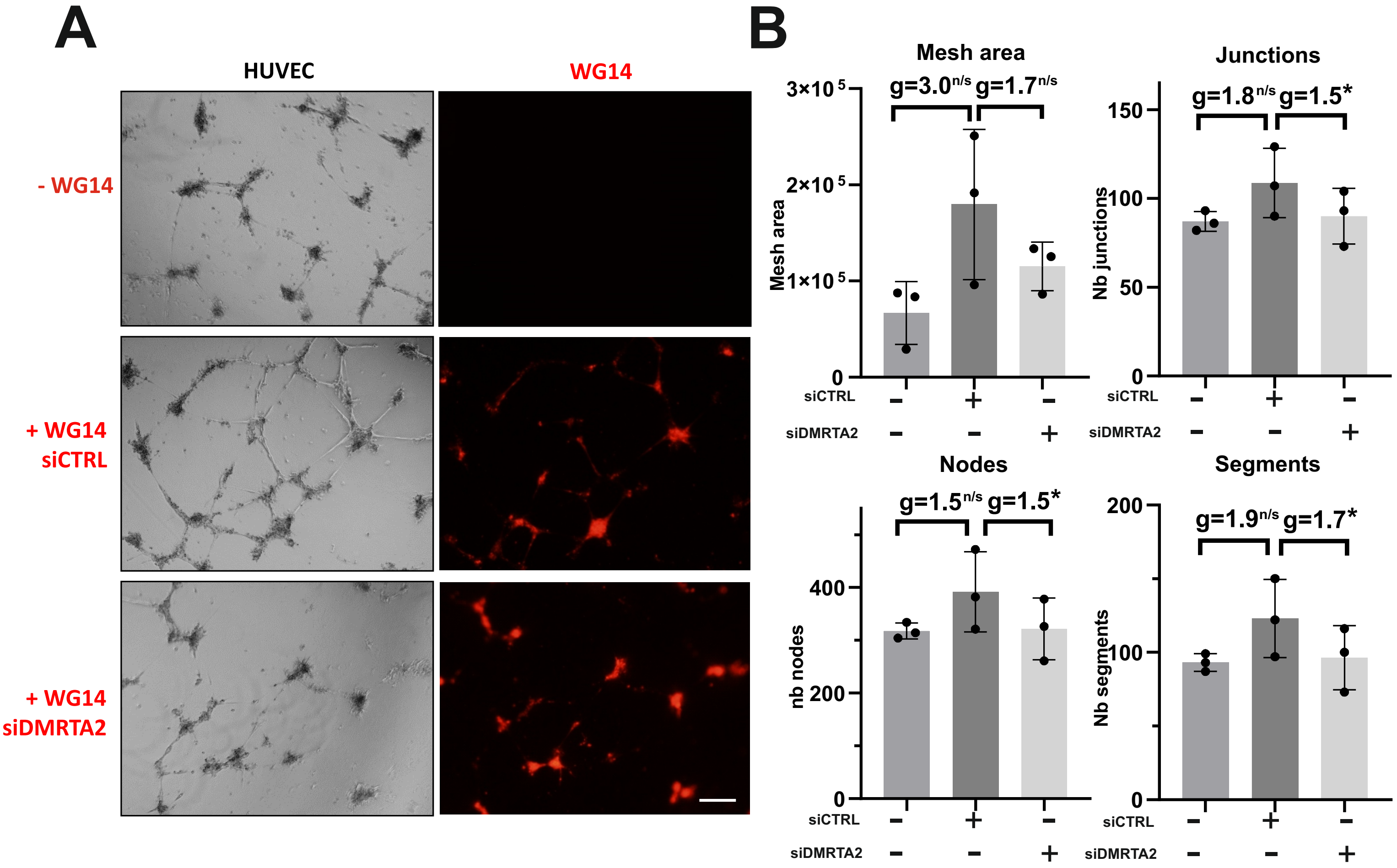

Supplemental figure 8. DMRTA2 is indispensable for WG14 primary glioma stem cells driven support of angiogenesis. (A) Representative photos of vascular nets formed by HUVEC alone or in co-culture with control WG14 cells or cells depleted of DMRTA2, scale bar – 200 µm. (B) Quantification of selected properties of the vascular net formed by HUVEC alone or in co-culture with control or DMRTA2 depleted WG14 cells. * - $p < 0.05$; ** - $p < 0.01$; *** - $p < 0.001$. Hedge's 'g' stands for effect size.
