# Supplementary figures and images for: DMRTA2 supports glioma stem-cell mediated neovascularization in glioblastoma
Source: Cell Death Dis. 2024 Mar 20;15(3):228. doi: 10.1038/s41419-024-06603-y (PMC10954651; doi:10.1038/s41419-024-06603-y)

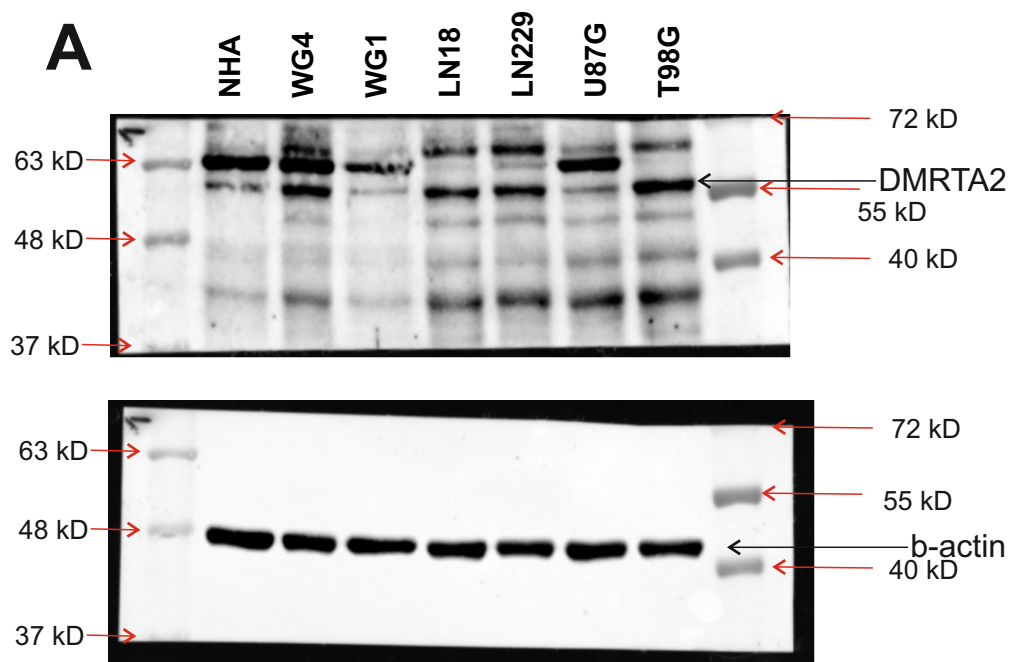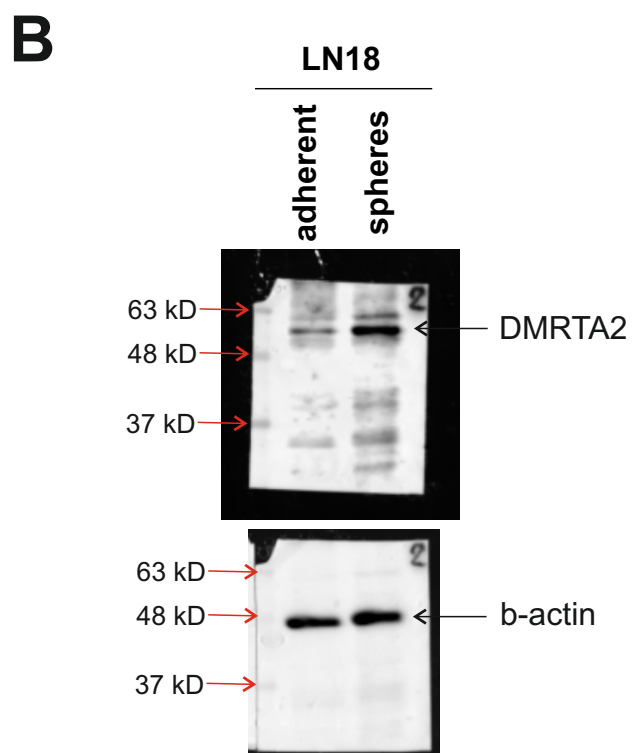

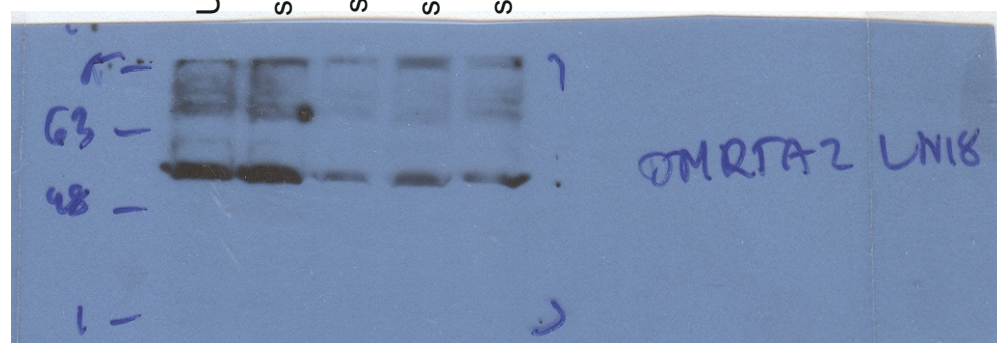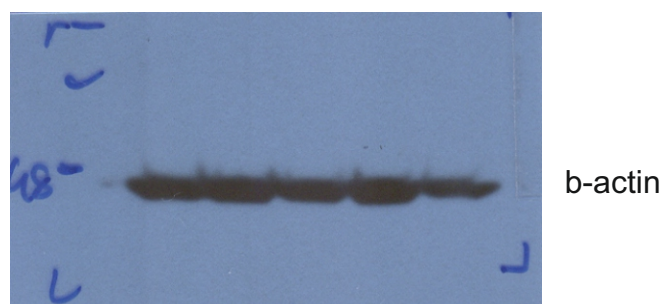

Supplement: Supplementary file 2 — Original Data File [file 41419_2024_6603_MOESM2_ESM.pdf]
